# Translation, Cross-Cultural Adaptation and Preliminary Validation of the Italian Version of the Early Cognitive and Listening Links (EarlyCaLL) Framework

**DOI:** 10.3390/audiolres16050160

**Published:** 2026-10-19

**Authors:** Valeria Caragli, Elisabetta Genovese, Daniele Monzani, Davide Soloperto, Gian Maria Galeazzi, Luca Pingani

**Affiliations:** 1 Audiology Program, Otorhinolaryngology Unit, Department of Medical and Surgical Sciences for Children and Adults, University of Modena and Reggio Emilia, 41124 Modena, Italy; davide.soloperto@unimore.it; 2 Audiology Program, Department of Maternal, Child and Adult Medical and Surgical Sciences, University of Modena and Reggio Emilia, 41124 Modena, Italy; elisabetta.genovese@unimore.it; 3 ENT and Audiology Department, University Hospital of Verona, 37126 Verona, Italy; daniele.monzani@univr.it; 4 Department of Biomedical, Metabolic and Neural Sciences, University of Modena and Reggio Emilia, 41124 Modena, Italy; gianmaria.galeazzi@unimore.it (G.M.G.); lpingani@unimore.it (L.P.); 5 Department of Integrated Mental Health and Substance Abuse Services, Azienda USL—IRCCS of Reggio Emilia, 42122 Reggio Emilia, Italy

**Keywords:** cochlear implant, complex needs, communication, EarlyCaLL, validation, cross-cultural adaptation

## Abstract

**Background/Objectives:** Children with severe-to-profound hearing loss and additional disabilities, receiving cochlear implant (CI), require multidimensional outcome measures that extend beyond conventional assessments of auditory perception and spoken language. The Early Cognitive and Listening Links (EarlyCaLL) framework was originally developed by the Nottingham Auditory Implant Programme team, in the United Kingdom, led by Datta and colleagues, to support expectation counselling and to monitor post-cochlear implantation progress in children with CI and associated complex needs. The framework was designed to capture broader developmental outcomes, including cognition, social and emotional engagement, listening and understanding, vocalisations and early talk, and augmentative and alternative communication (AAC), alongside CI equipment use and signal tolerance. However, to date, no validated Italian version of the Framework has been available. This study aims to translate, culturally adapt, and perform a preliminary psychometric validation of the Italian version of the EarlyCaLL. **Methods:** A methodological study was conducted following international guidelines for translation and cross-cultural adaptation. The development phase included forward translation, expert committee review, back-translation, developer review, and pre-validation refinement. The validation phase involved 20 experienced professionals. **Results:** The expert panel demonstrated extensive clinical experience, with a 100% response rate. Face validity was excellent across all six domains (≥90% adequate ratings; Gwet’s AC1 = 0.82). Ninety percent of experts considered the questionnaire acceptable without substantial modifications, and 10% recommended only minor revisions. All 25 descriptors achieved perfect content validity, while the overall scale indices also indicated unanimous agreement regarding the relevance and adequacy of the instrument. **Conclusions:** Our results support the face and content validity of the Italian version of the EarlyCaLL, supporting further clinical validation as a culturally adapted multidimensional observational instrument for evaluating rehabilitation outcomes in children with CI and additional disabilities. The framework provides a comprehensive assessment of functional development and may facilitate individualized rehabilitation, multidisciplinary collaboration, and family-centred care in pediatric CI.

## 1. Introduction

Approximately 20–40% of children with severe-to-profound hearing loss present with one or more additional disabilities, including intellectual disability, autism spectrum disorder, cerebral palsy, developmental delay, visual impairment, or genetic syndromes [1,2,3,4]. These comorbidities create a highly heterogeneous clinical population in whom hearing loss coexists with different impairments affecting cognition, communication, motor abilities, adaptive functioning, and social participation. This condition makes both CI candidacy assessment and outcome evaluation considerably more complex than in children with isolated hearing loss.

Historically, the presence of additional disabilities was often considered a relative contraindication to pediatric CI because of concerns that these children would derive limited benefit from auditory stimulation and would not achieve satisfactory speech and language outcomes [2,3,4]. However, over the past two decades, accumulating evidence has substantially changed this perspective: although children with additional disabilities generally demonstrate slower developmental trajectories and lower absolute performance compared to peers with isolated hearing loss, CI consistently provides meaningful benefits. Specifically, these improvements do not concern only auditory and language skills but also environmental sound awareness, communicative intent, adaptive behaviour, social interaction, emotional engagement, quality of life, and family functioning [1,2,4,5]. Consequently, the benefit of CI in this population should not be evaluated exclusively based on spoken language acquisition but rather on broader developmental and functional outcomes that enhance participation in everyday life [1,2,3].

Despite this paradigm shift, outcome assessment in pediatric CI continues to rely predominantly on instruments originally developed and validated for children with isolated hearing loss. In this regard, conventional measures—including speech perception tests, language assessments, Categories of Auditory Performance (CAP), Speech Intelligibility Rating (SIR), and similar parent-reported questionnaires—primarily evaluate auditory and linguistic performance while assuming relatively homogeneous developmental trajectories [6,7,8]. As a consequence, children with additional disabilities are almost inevitably compared with normative developmental expectations that do not adequately reflect their clinical characteristics or developmental potential. This approach underestimates the real benefits of CI, as meaningful improvements in adaptive behaviour, social participation, emotional engagement, communicative intent, and quality of life often remain undetected [1,2,9].

Thus, the use of a multidimensional assessment tool is strongly recommended in order to globally evaluate rehabilitation outcomes beyond hearing and spoken language alone. Such an approach is also consistent with contemporary biopsychosocial models of disability and with family-centred rehabilitation, which emphasize individualized functional progress rather than comparison with normative developmental milestones.

Within this framework, the Nottingham Early Cognitive and Listening Links (EarlyCaLL) represents one of the few instruments specifically designed for children with hearing loss and severe or profound additional disabilities. Indeed, the EarlyCaLL questionnaire was specifically developed by the Nottingham Auditory Implant Programme to address the limitations of conventional outcome measures in children with hearing loss and severe or profound additional disabilities [9]. Rather than functioning as a normative developmental scale, EarlyCaLL is an observational, criterion-referenced framework designed to monitor incremental developmental changes over time, support individualized expectation counselling, and facilitate multidisciplinary clinical decision-making. Specifically, it evaluates six interconnected developmental domains: cochlear implant use and signal tolerance, cognition, social-emotional engagement, listening and comprehension, vocalizations and early spoken communication, and augmentative and alternative communication (AAC). By simultaneously assessing these domains, EarlyCaLL acknowledges that successful CI cannot be adequately described solely through speech perception or expressive language outcomes but instead reflects the dynamic interaction between auditory access, cognitive development, communication abilities, adaptive functioning, and participation in everyday life [9,10].

Moreover, unlike traditional assessment batteries, EarlyCaLL adopts an individualized developmental perspective, documenting clinically meaningful progress even when children do not achieve age-expected language milestones. This characteristic is particularly relevant for children with complex neurodevelopmental conditions, who frequently develop alternative communication strategies, demonstrate improvements in attention, social reciprocity, environmental awareness, emotional engagement, or adaptive behaviour, while exhibiting relatively limited gains in spoken language. Consequently, the framework shifts the focus from normative comparisons toward the identification of functional changes that are meaningful for both the child and the family, providing clinicians with a more realistic evaluation of rehabilitation outcomes and supporting personalized intervention planning [2,9].

Finally, the original validation demonstrated that the earlyCaLL successfully captured developmental improvements that were not detected by conventional auditory or language measures [9].

Given the absence of a validated Italian version of this instrument as well as its potential in clinical practise, the present study aimed to translate, culturally adapt, and preliminarily validate the EarlyCaLL framework. By providing a standardized multidimensional assessment tool, this study seeks to facilitate more comprehensive evaluation of children with hearing loss and additional disabilities and to support individualized, multidisciplinary, and family-centred rehabilitation following CI.

## 2. Materials and Methods

### 2.1. Study Design

The protocol of this study consisted of two main phases:

1. Development Phase (translation and cultural adaptation)
2. Validation Phase (face validity and content validity)

### 2.2. Development Phase

During this phase, two independent native Italian translators (VC, DM)—both with professional experience in clinical audiology and linguistic adaptation—produced forward translations of the original EarlyCaLL. Translators were instructed to prioritize conceptual equivalence, ensuring that terminology related to communication behaviours, device use, and developmental constructs reflected Italian clinical practice.

Written permission was obtained from NUH NHS Trust and the Nottingham Auditory Implant Programme, as the copyright holders of the original EarlyCaLL materials, to translate, culturally adapt, and publish the Italian version of the instrument, including the original English files (S2, S4, S6, S8, S10) as Supplementary Material.

A multidisciplinary committee composed of otorhinolaryngologists and speech-language pathologists (EG, DS) synthesized the forward translations. The committee reviewed semantic, idiomatic, and experiential equivalence, ensuring that each item was understandable for Italian clinicians and caregivers of varied educational backgrounds. Discrepancies were resolved through consensus, resulting in a unified preliminary Italian version.

A bilingual translator unfamiliar with EarlyCaLL and not involved in previous steps performed a back-translation into English (GMG). The research team compared this version with the original to detect discrepancies or potential conceptual distortions. No significant inconsistencies were identified.

The reconciled Italian version, accompanied by translation documentation, was submitted to the original developers of EarlyCaLL. Minor modifications—mainly related to wording simplification and examples—were incorporated before initiating full validation.

### 2.3. Validation Phase

An expert team panel, consisting of 20 professionals, was selected to perform the validation phase, on the basis of the specific expertise relevant to the instrument. Specifically, 10 speech-language pathologists and 10 otolaryngologists/audiologists were identified. All participants were actively involved in the rehabilitation of hearing loss and/or the diagnostic, surgical, and audiological management of patients with hearing impairment. This panel composition was intentionally designed to ensure that validity judgments were provided by professionals with direct expertise in the clinical constructs assessed by the EarlyCaLL, rather than by experts with more general clinical experience. No patients or patient-related data were involved, and no clinical procedures or interventions were performed. Therefore, formal approval by an Ethics Committee/Institutional Review Board was not required, in accordance with the applicable institutional requirements. All participating healthcare professionals provided written informed consent to participate in the validation process.

A preliminary psychometric validation study was conducted to assess the face validity and content validity of the Italian version of the EarlyCaLL questionnaire. Consistent with methodological recommendations for the cross-cultural adaptation of clinical instruments [11], the validation process was structured into two complementary phases: (1) face validity, evaluating the comprehensibility, clarity, and clinical applicability of the Italian version; and (2) content validity, evaluating the relevance and necessity of the instrument’s content in relation to the constructs it is intended to measure.

### 2.4. Data Collection Instruments

#### 2.4.1. Face Validity Questionnaire

For each of the six EarlyCaLL domains (S1—Cochlear implant use/signal tolerance and integration; S2—Cognition; S3—Social and emotional engagement; S4—Listening and comprehension; S5—Vocalizations and early words; S6—Augmentative and Alternative Communication [AAC]), experts were asked to rate four aspects using a 3-point Likert scale (1 = inadequate; 2 = partially adequate; 3 = adequate): text comprehensibility, descriptor clarity, terminology appropriateness, and clinical applicability. Free-text comments were also collected for each domain. An additional general section (GEN) assessed the overall comprehensibility of the instrument, appropriateness of the language, usability within the Italian clinical context, and clarity of the instructions. Finally, experts provided an overall acceptability rating on a 4-point scale (1 = yes, without substantial modifications; 2 = yes, with minor modifications; 3 = yes, but with major modifications; 4 = no, substantial revision required).

#### 2.4.2. Content Validity Questionnaire

The same six domains included a total of 25 specific descriptors/items (4–5 per domain). Experts classified each item as essential, useful but not essential, or not necessary, following the procedure underlying the Content Validity Ratio (CVR) proposed by Lawshe [12]. In addition, each item was rated for relevance using a 4-point Likert scale (1 = not relevant; 2 = somewhat relevant; 3 = quite relevant; 4 = highly relevant), forming the basis for the Content Validity Index (CVI) according to Polit and Beck [13]. Four additional criteria evaluating domain coverage and the overall coherence of the instrument were assessed using the same 4-point relevance scale only.

The two questionnaires were distributed to the same panel of 20 clinical experts.

### 2.5. Statistical Analysis

For face validity, descriptive statistics were calculated for each domain and evaluation criterion, including mean, standard deviation, median, the percentage of ratings classified as adequate, and the cumulative percentage of ratings classified as adequate or partially adequate. Face validity was considered acceptable when ≥80% of experts rated a criterion as adequate [14,15]; the cumulative percentage of adequate or partially adequate ratings was reported as a secondary descriptive index. Inter-rater agreement was assessed using the proportion of experts selecting the modal category, pairwise observed agreement, Fleiss’ kappa, and Gwet’s AC1 with 95% confidence intervals, treating each evaluation criterion as a rated subject. AC1 was included because kappa is unstable when ratings are concentrated in a single category, whereas AC1 is less sensitive to skewed distributions [16,17]. Agreement indices were interpreted as measures of expert consensus rather than reliability [18,19], and AC1 values were benchmarked according to Landis and Koch [20].

For content validity, the Content Validity Ratio (CVR) was calculated for each item using the formula:

CVR = (n_e_ − N/2)/(N/2),

where n_e_ is the number of experts rating the item as essential and N = 20. According to Lawshe [12], the minimum acceptable CVR for a panel of 20 experts is 0.42. The Item-Level Content Validity Index (I-CVI) was computed as the proportion of experts assigning a relevance rating of 3 or 4, with values ≥0.78 considered acceptable [20].

At the scale level, two summary indices were calculated: the Scale-Level Content Validity Index based on the Average method (S-CVI/Ave), corresponding to the mean of all I-CVI values, and the Scale-Level Content Validity Index based on Universal Agreement (S-CVI/UA), representing the proportion of items achieving unanimous agreement among experts. All statistical analyses were performed using Python version 3.13.14 (pandas and stats models).

## 3. Results

### 3.1. Expert Panel

The panel consisted of 20 professionals, whose clinical experience ranged from 10 to 45 years (mean = 24.3 years, SD = 10.0). Nineteen of the twenty experts (95%) reported excellent experience in working with children with cochlear implants, while one expert (5%) reported good experience. In addition, 14 experts (70%) reported excellent experience in managing children with complex disabilities, communication disorders, or AAC, whereas the remaining six experts (30%) reported good experience.

All 20 recruited experts completed both questionnaires, yielding a response rate of 100%.

### 3.2. Face Validity

Expert evaluation indicated excellent face validity and comprehensibility of the translated questionnaire. Across the six domains, mean scores ranged from 2.90 (SD = 0.31) to 3.00 (SD = 0.00), with a median score of 3 for all sections. Five domains achieved an adequacy rate of 90%, while the AAC domain reached 100% adequacy. All domains therefore met the predefined ≥80% adequacy criterion. Table 1 reports the scores for each EarlyCaLL domain. Importantly, all domains obtained 100% of ratings classified as either adequate or partially adequate. Likewise, evaluation of the questionnaire’s overall characteristics demonstrated excellent performance, with mean scores of 2.90 for overall comprehensibility, language appropriateness, and clarity of instructions, and 3.00 for usability. Each general criterion was rated as adequate by at least 90% of experts. All questionnaire characteristics were judged as either adequate or partially adequate by 100% of the experts. Table 2 summarizes the results for the general section (GEN).

The questionnaire was overall acceptable: eighteen of the 20 experts (90%) judged the instrument as “acceptable without substantial modifications,” whereas the remaining two experts (10%) rated it as “acceptable with minor modifications”. No expert indicated that major modifications or substantial revision were required.

The proportion of experts selecting the modal category ranged from 90% to 100% across criteria, and pairwise observed agreement was 84.4%. Fleiss’ kappa was close to zero (κ = −0.03), a pattern consistent with the kappa paradox [21], which arises when ratings are highly concentrated in one category. Gwet’s AC1, which is robust to this paradox, indicated almost perfect agreement (AC1 = 0.82, 95% CI 0.78–0.86; domains: 0.81, 0.77–0.86; general section: 0.84, 0.63–1.00). Lower ratings were given by two experts who systematically rated all criteria of domains S1–S5 as partially adequate, indicating a rater-level rather than item-level source of disagreement. Both of these experts reported excellent experience with children with CI and with complex disabilities/AAC, whereas all experts reporting good experience in either area (N = 1 and N = 6, respectively) rated every criterion as adequate. Content validity ratings were identical across all experts, irrespective of experience level.

Two experts (10%) consistently reported, across all six domains, some redundancy between the descriptions/examples provided in the scoring forms and the corresponding appendices containing the administration instructions. Within the general section, additional suggestions included harmonizing the terminology used throughout the instrument, clarifying the meaning of the term signal, and arranging the items in ascending order of difficulty.

### 3.3. Content Validity

All 25 descriptors achieved a Content Validity Ratio (CVR) of 1.00, as all 20 experts rated every descriptor as essential, and an Item-Level Content Validity Index (I-CVI) of 1.00, as all experts rated every descriptor as quite or highly relevant. Inspection of the raw data showed that all relevance ratings were 4 (highly relevant), yielding a mean relevance score of 4.00 for each descriptor. No descriptor fell below either the critical Lawshe CVR value for a panel of 20 experts (0.42) or the recommended I-CVI acceptability threshold (0.78). Item-level results are reported in Supplementary Table S1.

Consequently, the scale-level indices also demonstrated perfect content validity, with S-CVI/Ave = 1.00 and S-CVI/UA = 1.00, reflecting unanimous agreement across all 25 items. Gwet’s AC1 was 1.00; Fleiss’ kappa was not computable, as all ratings fell into a single category.

Similarly, the four global evaluation criteria (coverage of relevant domains, consistency with the intended clinical objectives, absence of missing domains, and adequacy of the longitudinal structure) all achieved a mean score of 4.00/4, with unanimous agreement among the experts.

No free-text comments or suggestions were provided regarding the addition, removal, or modification of any content.

## 4. Discussion

The present study represents the first Italian cross-cultural adaptation and preliminary validation of the Early Cognitive and Listening Links (EarlyCaLL), a multidimensional observational framework, specifically developed for children with cochlear implants and additional disabilities.

Although CI has dramatically improved auditory access and spoken language development for children with severe-to-profound hearing loss, predicting and evaluating outcomes in children with comorbidities and special needs remain considerably more challenging [2,22,23,24]. Indeed, these conditions substantially influence auditory development, cognitive maturation, communication skills, adaptive behaviour, and social participation, making conventional audiological outcome measures only partially representative of the true benefits achieved after CI. Nonetheless, the current use of traditional assessment batteries is not fully adequate in patients with CI and AD, as these tools were primarily developed for children with isolated hearing loss, assuming relatively homogeneous developmental trajectories [6,7,8]. Moreover, most of these batteries evaluate specific auditory or linguistic skills, rather than providing a comprehensive assessment of the child’s overall developmental profile. Consequently, reliance exclusively on auditory perception or expressive language outcomes may substantially underestimate the functional impact of cochlear implantation in children with complex developmental conditions.

Within this context, the principal strength of EarlyCaLL lies in the conceptual model on which it is built. This multidimensional organization recognizes that successful rehabilitation in children with additional disabilities cannot be adequately described by auditory performance alone but instead reflects the dynamic interaction between sensory input, cognitive development, communication strategies, adaptive functioning, and participation in everyday life [2,9].

Our findings demonstrated excellent face and content validity of the Italian EarlyCaLL, with high agreement among experienced audiologists and speech-language pathologists on clarity, comprehensibility, and applicability, and unanimous agreement on the clinical relevance of all evaluated domains. Moreover, our preliminary data also support the subsequent clinical and psychometric evaluation of EarlyCaLL. Indeed, our results suggest that the Italian version faithfully preserves the conceptual integrity of the original framework while ensuring linguistic, semantic, and cultural appropriateness for clinical use within Italian pediatric audiological rehabilitation services [9]. The significance of these findings extends well beyond the linguistic adaptation of an existing assessment tool. Rather, they address one of the most important unmet needs in pediatric CI: the availability of outcome measures capable of capturing the complexity of rehabilitation in children presenting with additional disabilities.

This conceptual approach represents a substantial shift from the traditional auditory-centred paradigm, following the child’s overall developmental trajectory. Such an individualized perspective is particularly appropriate because children with complex developmental disorders frequently develop alternative communication strategies, rely on multimodal interaction, or achieve meaningful functional improvements without necessarily reaching age-expected spoken language milestones. In this regard, the inclusion of augmentative and alternative communication constitutes one of the framework’s most innovative aspects. Historically, AAC has often been considered an alternative reserved for children unable to develop spoken language. Contemporary rehabilitation models, however, increasingly recognize AAC as an integral component of multimodal communication that facilitates language acquisition, reduces communicative frustration, enhances social participation, and supports overall developmental progress. By incorporating AAC as one of its developmental domains, EarlyCaLL acknowledges that successful hearing rehabilitation should ultimately be defined by effective communication rather than by exclusive spoken language acquisition [10]. Consequently, the framework shifts the focus from normative comparisons toward individualized developmental progress, an approach increasingly advocated within pediatric rehabilitation and family-centred care [10].

The multidimensional structure of EarlyCaLL is also highly consistent with the biopsychosocial model (ICF) [25], conceptualizing disability as the result of interactions among body functions and structures, activities, participation, environmental influences, and personal factors, rather than as the direct consequence of a medical diagnosis alone [19]. Beyond outcome measurement, the framework also offers important clinical implications for rehabilitation planning and expectation counselling. Indeed, establishing realistic expectations before CI remains one of the greatest challenges when counselling families of children with complex developmental conditions. Prognostic uncertainty may generate anxiety among caregivers, leading either to unrealistic expectations or unnecessary therapeutic pessimism. The original Nottingham group designed EarlyCaLL specifically to also facilitate individualized expectation counselling by allowing clinicians and families to visualize incremental developmental changes across multiple domains throughout rehabilitation. By emphasizing meaningful improvements in everyday functioning, rather than focusing exclusively on speech acquisition, the framework supports shared clinical decision-making and family-centred care [10]. Moreover, repeated administration of EarlyCaLL may facilitate individualized rehabilitation planning by identifying domain-specific strengths and weaknesses requiring targeted intervention.

Finally, as the questionnaire simultaneously evaluates multiple domains, it naturally promotes multidisciplinary collaboration among otolaryngologists, audiologists, speech-language pathologists, psychologists, child neuropsychiatrists, rehabilitation therapists, educators, and families [2]. Furthermore, the increasing adoption of electronic health records and digital outcome monitoring systems creates opportunities for integrating EarlyCaLL into routine clinical practice, enabling longitudinal visualization of developmental trajectories and supporting data-driven therapeutic decision-making.

Unlike most patient-reported or performance-based outcome measures, the EarlyCaLL framework was specifically developed for children with cochlear implants and severe additional disabilities, a highly heterogeneous population characterized by complex neurodevelopmental profiles, diverse communication abilities, and markedly variable developmental trajectories. Because of this clinical complexity, conventional psychometric validation strategies based on large homogeneous patient cohorts are difficult to implement and may not adequately reflect the intended purpose of the instrument. Accordingly, the present preliminary validation was intentionally designed in accordance with internationally accepted recommendations for the cross-cultural adaptation of clinical assessment tools, where expert evaluation of face and content validity represents the essential first step before broader psychometric testing.

A further strength lies in the rigorous methodological approach adopted throughout the translation and adaptation process, the results of which support the subsequent clinical use and psychometric evaluation of the questionnaire.

Forward translation, multidisciplinary expert review, back-translation, evaluation by the original developers, and pre-validation refinement ensured conceptual, semantic, and cultural equivalence between the Italian version and the original framework. Importantly, the validation panel was deliberately composed of clinicians with extensive expertise in pediatric CI, auditory rehabilitation, AAC, and complex neurodevelopmental disabilities. This choice was particularly relevant because the clinical constructs evaluated by EarlyCaLL require highly specialized knowledge and cannot be adequately judged by professionals without direct experience in this patient population.

The high level of agreement observed among experts further supports the robustness of the adapted instrument. All descriptors achieved perfect Content Validity Ratio (CVR = 1.00) and Item-Level Content Validity Index (I-CVI = 1.00), while both scale-level indices also reached 1.00, demonstrating agreement regarding the relevance and representativeness of every domain. Similarly, face validity demonstrated excellent comprehensibility, clarity, and clinical applicability across all sections. The concentration of ratings in a single category produced the kappa paradox, limiting the interpretability of kappa; the chance-corrected AC1 coefficient (0.82) confirmed almost perfect agreement among experts.

In regard to this study, several limitations should also be acknowledged. First, this study represents a preliminary psychometric validation focused on translation, cultural adaptation, face validity, and content validity. Additional measurement properties—including construct validity, test–retest reliability, responsiveness to longitudinal change, and criterion validity—remain to be investigated in future studies. Second, the validation relied exclusively on expert assessment and did not include direct administration of the Italian EarlyCaLL to children and their families. This methodological choice was deliberate, as establishing conceptual and content equivalence is considered a prerequisite before evaluating the instrument in clinical populations, particularly when the target population presents profound heterogeneity and complex disabilities. Accordingly, the agreement indices reported here reflect expert consensus on the instrument’s content and wording, not the inter-rater reliability of EarlyCaLL scoring, which should be established when the tool is administered to children. In addition, the uniformly maximal content validity ratings suggest a possible ceiling effect, which limits the ability of these indices to discriminate between items. A further limitation is that the face and content validity questionnaires were administered to the same experts at the same time, which may have introduced respondent burden and a halo effect, whereby a positive overall impression of the translation may have inflated content validity ratings and contributed to the uniformly high scores observed; future studies should consider administering the two assessments separately or in counterbalanced order. Moreover, caregivers/parents were not directly involved in the cultural adaptation phase, despite the family-centred approach of the EarlyCaLL framework, and their perspectives should therefore be incorporated in future validation studies. Finally, although the expert panel included professionals with extensive experience and achieved complete response rates, all participants were recruited within the Italian healthcare system, and further multicentre studies will be required to confirm the generalizability of the findings across different clinical settings.

Future research should therefore focus on the clinical validation of the Italian EarlyCaLL in larger cohorts of children with cochlear implants and additional disabilities, evaluating its longitudinal responsiveness, inter-rater reliability, construct validity, and ability to detect clinically meaningful developmental changes during rehabilitation. Such studies will provide further evidence supporting its use as a standardized multidimensional outcome measure for both routine clinical practice and research.

## 5. Conclusions

This study provides the first evidence supporting the face and content validity of the Italian version of the EarlyCaLL framework. Unlike conventional audiological outcome measures that predominantly focus on auditory perception or spoken language, EarlyCaLL adopts a multidimensional developmental perspective encompassing cochlear implant use, cognition, listening, communication, socio-emotional functioning and AAC. This comprehensive approach reflects the contemporary biopsychosocial model of disability and responds to the growing recognition that meaningful rehabilitation outcomes in children with cochlear implants—particularly those with additional disabilities—extend well beyond speech perception alone. The present findings represent an important step toward the implementation of a standardized, culturally adapted and clinically meaningful observational framework capable of supporting individualized rehabilitation, multidisciplinary collaboration and family-centred care in pediatric CI, supporting its use in clinical practise.

## Figures and Tables

**Table 1:** Face validity scores for each EarlyCaLL domain (N = 20 experts); Ratings: 1 = inadequate, 2 = partially adequate, 3 = adequate. Acceptability criterion: ≥80% “adequate”.

Section	Mean (SD)	Median	“Adequate”, % (N)	“Adequate” + “Partially Adequate”, % (N)
S1—Cochlear implant use/signal tolerance	2.90 (0.31)	3	90% (18/20)	100% (20/20)
S2—Cognition	2.90 (0.31)	3	90% (18/20)	100% (20/20)
S3—Social and emotional engagement	2.90 (0.31)	3	90% (18/20)	100% (20/20)
S4—Listening and comprehension	2.90 (0.31)	3	90% (18/20)	100% (20/20)
S5—Vocalizations and early words	2.90 (0.31)	3	90% (18/20)	100% (20/20)
S6—Augmentative and alternative communication (AAC)	3.00 (0.00)	3	100% (20/20)	100% (20/20)

**Table 2:** Face validity scores for the general section (GEN) (N = 20 experts); Ratings: 1 = inadequate, 2 = partially adequate, 3 = adequate. Acceptability criterion: ≥80% “adequate”.

Item	Mean (SD)	Median	“Adequate”, % (N)	“Adequate” + “Partially Adequate”, % (N)
Overall comprehensibility	2.90 (0.31)	3	90% (18/20)	100% (20/20)
Appropriateness of language	2.90 (0.31)	3	90% (18/20)	100% (20/20)
Usability in Italy	3.00 (0.00)	3	100% (20/20)	100% (20/20)
Clarity of instructions	2.90 (0.31)	3	90% (18/20)	100% (20/20)

## Data Availability

The data supporting the findings of this study are available from the corresponding author upon reasonable request.

## Supplementary Materials

The following supporting information can be downloaded at: https://www.mdpi.com/article/10.3390/audiolres16050160/s1, Supplementary Material S1: including Instructions ITA; Supplementary Material S2: including Instructions EN; Supplementary Material S3: including Use of IC ITA; Supplementary Material S4: including Use of IC EN; Supplementary Material S5: including Schedule ITA; Supplementary Material S6: including Schedule EN; Supplementary Material S7: including Schedule Instructions ITA; Supplementary Material S8: including Schedule Instructions EN; Supplementary Material S9: including Summary ITA; Supplementary Material S10: including Summary EN; Supplementary Material S11: including Table S1: Item-level content validity of the Italian EarlyCaLL (N = 20 experts).

## Abbreviations

The following abbreviations are used in this manuscript:

CI	Cochlear Implant
AAC	Augmentative Alternative Communication
