# Supplementary material for: Translation, Cross-Cultural Adaptation and Preliminary Validation of the Italian Version of the Early Cognitive and Listening Links (EarlyCaLL) Framework
Source: Audiol Res. 2026 Oct 19;16(5):160. doi: 10.3390/audiolres16050160 (PMC13701395; doi:10.3390/audiolres16050160)
Supplement: Supplementary file 1 [file audiolres-16-00160-s001.zip › supplementary S1.pdf]

Nottingham Auditory Implant Programme

Nottingham University Hospitals NHS Trust

Appendice 1

Early Cognitive and Listening Links: Early CaLL

EQUIPMENT USE, SIGNAL TOLERANCE AND INTEGRATION (Revised January 2019)

Nome:	DOB:	Intervallo di tempo:	NHS No:
TOD/SLT:	Firma:		Data:

√	TOLLERANZA/USO DEL SEGNALE
5	**Rapporto positivo ed uso costante.** Ha instaurato un rapporto positivo, sia consciamente che incosciamente con l'apparecchiatura ed il segnale uditivo. Es: può chiedere di utilizzare l' IC, di rimetterlo se rimosso o se non utilizzabile, indicarne un malfunzionamento.
4	**Significativi cambiamenti comportamentali** quando il dispositivo viene indossato, a dimostrazione di un uso funzionale del segnale, durante la gran parte delle ore di veglia.
3	**Chiari cambiamenti comportamentali** quando il dispositivo è indossato, seppur solo in presenza di forte motivazione all'ascolto di suoni/voci e senza attività strutturate. Tipicamente, uso quotidiano/regolare, ma tendenzialmente solo in parte del tempo.
2	**Passiva** accettazione del dispositivo e del segnale. *Non* evidenti cambiamenti comportamentali. Possono esserci periodi di uso esteso (es anche tutte le ore di veglia).
1	**Uso Limitato** - brevi periodi - e continui episodi di non utilizzo per non tolleranza del segnale. Può tenerlo solo quando è distratto o occupato dagli adulti.
0	**Nessuno/poco uso** - il dispositivo viene rifiutato o attivamente rimosso. Reazioni avverse al segnale.

TEMPO DI USO			
Numero solito di ore d'uso del processore al giorno	Ore	Numero solito di ore di veglia al giorno	Ore

FATTORI CHE INFLUENZANO IL TEMPO DI USO<br>Per favore indicare con √ qualsiasi fattore rilevante	
□ **Nessuno** - Non ci sono fattori, diversi dalla tolleranza al segnale, che influenzino il tempo d'uso.	
□ Malattia severa e/o trattamento ospedaliero tale da rendere il bambino non in grado o non a proprio agio nell' indossare il dispositivo per un periodo di tempo esteso, ma solo limitato. *Es. Il bambino è stato molto malato in ospedale per una infezione alle vie aeree*	□ Malattia grave/cronica in corso o necessità di trattamenti regolari, come la fisioterapia, che comportano periodi fissi o prolungati in cui il bambino non indossa il dispositivo *Es: grave epilessia*
□ Il design della sedia a rotelle o della seduta speciale del bambino fa sì che la bobina e/o l'unità di elaborazione vengano spesso urtati, rendendo impraticabile l'uso dell'attrezzatura. Questo può essere un problema a breve o a lungo termine.	□ Il livello di sviluppo, le condizioni anatomiche o le difficoltà fisiche del bambino rendono difficile indossare il dispositivo in alcuni momenti o situazioni.<br>Ad esempio, il bambino ha bisogno di mettere un casco, in determinati momenti, con il quale è difficile indossare il dispositivo.
□ Reazione emotiva a un cambiamento nella routine o nella situazione familiare. *Es: nuovo insegnante a scuola*	☐ Altre priorità riportate dai genitori/care givers. (per favore elencare):<br>☐ Altro (per favore elencare):
Per favore commentare per esteso ciò che influenza l' uso del processore/i e qualsiasi possibile azione/soluzione:	

Copyright©NUH NHS Trust 2019. All rights reserved. Not to be reproduced in whole or part without the permission of the copyright owner.
