# Supplementary material for: Translation, Cross-Cultural Adaptation and Preliminary Validation of the Italian Version of the Early Cognitive and Listening Links (EarlyCaLL) Framework
Source: Audiol Res. 2026 Oct 19;16(5):160. doi: 10.3390/audiolres16050160 (PMC13701395; doi:10.3390/audiolres16050160)
Supplement: Supplementary file 1 [file audiolres-16-00160-s001.zip › Supplementary S10.pdf]

PATIENT DETAILS		
Name of Patient:	DOB:	NHS No:

RECORD OF PROGRESS										
Date of Assessment	Parent/Carer and other professionals contributing	Scores						Comments	Professional completing assessment (Print name)	Signature
		EQUIPMENT USE, SIGNAL TOLERANCE	COGNITION	SOCIAL & EMOTIONAL ENGAGEMENT	LISTENING, UNDERSTANDING	VOCALISATIONS, EARLY WORDS	AAC			

Copyright©NUH NHS Trust 2016. All rights reserved. Not to be reproduced in whole or part without the permission of the copyright owner
