# Supplementary material for: Translation, Cross-Cultural Adaptation and Preliminary Validation of the Italian Version of the Early Cognitive and Listening Links (EarlyCaLL) Framework
Source: Audiol Res. 2026 Oct 19;16(5):160. doi: 10.3390/audiolres16050160 (PMC13701395; doi:10.3390/audiolres16050160)
Supplement: Supplementary file 1 [file audiolres-16-00160-s001.zip › supplementary S11.pdf]

New Supplementary Table S1. Item-level content validity of the Italian EarlyCaLL (N = 20 experts)

**Domain**	**Descriptor**	**n essential (/20)**	**CVR**	**Relevance ratings 1/2/3/4 (n)**	**Mean relevance**	**I-CVI**
S1 – CI use/signal tolerance	Tolerance and acceptance of the device and the auditory signal	20	1.00	0/0/0/20	4.00	1.00
	Signal use: from absence/refusal to passive acceptance and regular use	20	1.00	0/0/0/20	4.00	1.00
	Behavioural changes indicating functional use of the signal	20	1.00	0/0/0/20	4.00	1.00
	Daily processor use time relative to waking hours	20	1.00	0/0/0/20	4.00	1.00
	Clinical, postural, environmental or family factors affecting use time	20	1.00	0/0/0/20	4.00	1.00
S2 – Cognition	Reflex behaviours, sensory exploration and recognition of faces/familiar people	20	1.00	0/0/0/20	4.00	1.00
	Attention, preferences and anticipation of familiar routines or activities	20	1.00	0/0/0/20	4.00	1.00
	Object permanence, cause–effect relationships and simple problem solving	20	1.00	0/0/0/20	4.00	1.00
	Functional/symbolic play, visual memory and early symbolic skills	20	1.00	0/0/0/20	4.00	1.00
S3 – Social and emotional engagement	Responses to adult initiations and regulation through the caregiver	20	1.00	0/0/0/20	4.00	1.00
	Active seeking of interaction/communication and differentiation of familiar people	20	1.00	0/0/0/20	4.00	1.00
	Eye contact, joint attention and participation in social games	20	1.00	0/0/0/20	4.00	1.00
	Emotional understanding, response to others' emotional states and participation in small groups	20	1.00	0/0/0/20	4.00	1.00

Domain	Descriptor	n essential (/20)	CVR	Relevance ratings 1/2/3/4 (n)	Mean relevance	I-CVI
S4 – Listening and comprehension	Sound detection and early reactions to sounds, voices or environmental noises	20	1.00	0/0/0/20	4.00	1.00
	Behavioural responses to motivating sounds, familiar voices or structured activities	20	1.00	0/0/0/20	4.00	1.00
	Emotionally meaningful listening and functional use of the signal in daily life	20	1.00	0/0/0/20	4.00	1.00
	Comprehension of simple words/requests and monitoring of the sound environment	20	1.00	0/0/0/20	4.00	1.00
S5 – Vocalisations and early words	Reflexive/involuntary sounds and early vocalisations	20	1.00	0/0/0/20	4.00	1.00
	Intentional vocalisations to attract attention, protest or express needs	20	1.00	0/0/0/20	4.00	1.00
	Babbling and imitation of sounds or vocal patterns	20	1.00	0/0/0/20	4.00	1.00
	First words/simple combinations and basic conversational exchanges	20	1.00	0/0/0/20	4.00	1.00
S6 – AAC	Non-verbal communication: gaze, gestures, pointing and body expressions	20	1.00	0/0/0/20	4.00	1.00
	Use/comprehension of objects of reference, photographs, pictures or symbols	20	1.00	0/0/0/20	4.00	1.00
	Use/comprehension of signs or visual systems shared with adults and peers	20	1.00	0/0/0/20	4.00	1.00
	Functional use of AAC, symbols or written words for requests, choices and daily exchanges	20	1.00	0/0/0/20	4.00	1.00
**Scale level**	S-CVI/Ave					1.00
	S-CVI/UA					1.00

Domain	Descriptor	n essential (/20)	CVR	Relevance ratings 1/2/3/4 (n)	Mean relevance	I-CVI
**Global criteria**	The instrument adequately covers the domains relevant to the target population	–	–	0/0/0/20	4.00	1.00
	Content is consistent with the clinical objectives of the EarlyCaLL	–	–	0/0/0/20	4.00	1.00
	No relevant domains are missing	–	–	0/0/0/20	4.00	1.00
	The overall structure is adequate for longitudinal assessment	–	–	0/0/0/20	4.00	1.00

Note. CVR = Content Validity Ratio (Lawshe); critical value for N = 20: 0.42. I-CVI = Item-level Content Validity Index (proportion of experts rating 3 or 4); acceptability threshold: 0.78. S-CVI/Ave = mean of I-CVIs; S-CVI/UA = proportion of items with universal agreement. Relevance scale: 1 = not relevant, 2 = somewhat relevant, 3 = quite relevant, 4 = highly relevant. Global criteria were rated on the relevance scale only.
