# Supplementary material for: Translation, Cross-Cultural Adaptation and Preliminary Validation of the Italian Version of the Early Cognitive and Listening Links (EarlyCaLL) Framework
Source: Audiol Res. 2026 Oct 19;16(5):160. doi: 10.3390/audiolres16050160 (PMC13701395; doi:10.3390/audiolres16050160)
Supplement: Supplementary file 1 [file audiolres-16-00160-s001.zip › supplementary S2.pdf]

Nottingham Auditory Implant Programme

Nottingham University Hospitals NHS
NHS Trust

Appendix One

Early Cognitive and Listening Links: Early CaLL

EQUIPMENT USE, SIGNAL TOLERANCE AND INTEGRATION (Revised January 2019)

Name:	DOB:	Interval:	NHS No:
TOD/SLT:	Signature:		Date:

√	TOLERANCE/USE OF THE SIGNAL
5	**Positive and consistent use.** Positive connection made, either consciously or unconsciously between the equipment and the signal delivered. eg may request use, get upset if removed or not available, indicate when there is a fault.
4	**Sustained behaviour changes** when the equipment is worn which demonstrate **functional use** of the signal for extended parts of the waking day.
3	**Definite behaviour changes** when equipment is worn although these may only be for highly motivating sounds/voices and within structured activities. Typically, daily/ regular use but may only be for part of the time.
2	**Passive** acceptance of the equipment and signal. Behaviour changes *not* evident. May have periods of extended use (ie as much as all waking hours).
1	**Limited** use - short periods - and continued episodes of non-use because signal is not tolerated. May only wear when distracted or occupied by adults.
0	**No/little use** - equipment is rejected or actively removed. Adverse reaction to the signal

WEARING TIME			
Number of hours per day processor/s typically worn	hours	Typical number of waking hours per day	hours

FACTORS AFFECTING WEARING TIME<br>Please indicate √ any factors are relevant	
☐ **None** - There are no factors, other than tolerance of the signal, that affect wearing time.	
☐ Severe illness and/or hospital treatment which meant that the child was not able or not comfortable to wear the equipment for an extended but definite period.<br>*E.g. The child was very ill in hospital with a chest infection*	☐ On-going severe/chronic illness or the need for regular treatments, such as physiotherapy, which result in regular or prolonged periods when the child does not wear the equipment.<br>*E.g. Severe Epilepsy*
☐ The design of the child's wheelchair or specialist seating causes the coil and/or processor to be knocked off frequently – making it impractical to wear the equipment. This may be a short term or long term issue.	☐ The child's stage of development, anatomy or physical difficulties makes it difficult for them to wear the equipment at certain times or in certain situations.<br>*E.g. The child needs to wear a helmet, at certain times, with which it is difficult to wear the equipment.*
☐ Emotional reaction to change in routine or family situation.<br>*E.g. New teacher at school*	☐ Other priorities reported by parent/carer. (please state):<br><br>☐ Other (please state):
Please comment on the extent to which this affects wearing of the processor/s and any possible actions/solutions:	

Copyright©NUH NHS Trust 2019. All rights reserved. Not to be reproduced in whole or part without the permission of the copyright owner.
