# Supplementary material for: Translation, Cross-Cultural Adaptation and Preliminary Validation of the Italian Version of the Early Cognitive and Listening Links (EarlyCaLL) Framework
Source: Audiol Res. 2026 Oct 19;16(5):160. doi: 10.3390/audiolres16050160 (PMC13701395; doi:10.3390/audiolres16050160)
Supplement: Supplementary file 1 [file audiolres-16-00160-s001.zip › supplementary S3.pdf]

Appendice uno

Punteggio	USO DEL DISPOSITIVO, TOLLERANZA E INTEGRAZIONE AL SEGNALE
5	**Buon adattamento e uso regolare.** Buona connessione, sia consapevole che inconscia, tra il dispositivo e il segnale fornito. Ad esempio, può richiedere l'uso del dispositivo, arrabbiarsi se viene rimosso o se non disponibile, indicare quando c'è un problema.<br>*Allo stadio Cinque il bambino monitora attivamente il proprio ambiente uditivo per lunghi periodi, può "richiedere" l'apparecchiatura o dimostrare consapevolezza dei cambiamenti nella qualità del segnale, sufficienti per influenzare il comportamento.*
4	**Cambiamenti di comportamento persistenti** quando il dispositivo viene applicato e che dimostrano un uso funzionale del segnale per lunghi periodi della giornata.<br>*Il passaggio alla quarta fase richiede che le capacità di attenzione generale del bambino e la capacità di interessarsi al mondo circostante siano sufficientemente sviluppate da poter monitorare in modo indipendente il loro ambiente uditivo per almeno una parte del tempo. Questa è una sfida enorme per i bambini PMLD o per quelli con una diagnosi aggiuntiva di autismo.*
3	**Cambiamenti di comportamento definiti** quando il dispositivo viene applicato, sebbene questo cambiamento possa essere limitato solo a suoni/voci di grande interesse e all'interno di attività strutturate. In genere, l'uso é quotidiano/regolare, ma potrebbe essere limitato a parte della giornata.<br>*Si tratta di un'ampia categoria che comprende i bambini in cui l'uso dell'elaboratore del suono può essere incoerente, ma per i quali il beneficio è evidente durante questi periodi, fino ai bambini che indossano l'apparecchiatura per la maggior parte delle loro ore di veglia, ma le cui capacità spontanee di monitoraggio uditivo rimangono limitate.*
2	**Accettazione passiva** del dispositivo e del segnale. I cambiamenti di comportamento non sono evidenti. Si possono avere periodi di uso prolungato (ad esempio fino a tutte le ore di veglia).<br>*L'apparecchiatura e il segnale sono accettati, ma non ci sono evidenti cambiamenti di comportamento. Non tutti i bambini attraversano questa fase e per altri potrebbe essere un periodo piuttosto breve. Tuttavia, per alcuni bambini in questa fase, il tempo in cui portare l'apparecchio potrebbe essere prolungato ed esteso alla maggior parte delle loro ore di veglia.*<br>*Questo accade perché l'accettazione passiva dell'apparecchiatura e del segnale non portano automaticamente alla capacitò di riconoscere le informazioni fornite come significative. Per molti bambini all'interno di questa popolazione, mantenere l'attenzione è uno sforzo e può essere raggiunto solo per periodi limitati per oggetti, persone e attività altamente motivanti.*
1	**Uso limitato - brevi periodi** - e continui episodi di non utilizzo perché il segnale non è tollerato. E' possibile che l'apparecchio sia indossato solo se il paziente è distratto o occupato in altre attività dagli adulti.<br>*L'uso delle apparecchiature è in genere per brevi periodi di tempo. In questa fase è probabile che il bambino indossi l'attrezzatura solo quando è altamente coinvolto dagli adulti, rassicurato e distratto. Senza questo, il bambino potrebbe provare a buttare giù la bobina ecc.*
0	**Nessun/poco uso** - l'apparecchio viene rifiutato o rimosso attivamente. Reazione avversa al segnale.<br>Per il momento non vi è alcun uso efficace. Il bambino è in difficoltà o cerca attivamente di rimuovere l'attrezzatura. La sensazione fisica del processore può essere tollerata, ma solo fino all'attivazione del segnale.
