# Supplementary material for: Translation, Cross-Cultural Adaptation and Preliminary Validation of the Italian Version of the Early Cognitive and Listening Links (EarlyCaLL) Framework
Source: Audiol Res. 2026 Oct 19;16(5):160. doi: 10.3390/audiolres16050160 (PMC13701395; doi:10.3390/audiolres16050160)
Supplement: Supplementary file 1 [file audiolres-16-00160-s001.zip › Supplementary S4.pdf]

Appendix one

Stage	**EQUIPMENT USE,SIGNAL TOLERANCE AND INTEGRATION**<br>*-additional guidance notes in italics*
5	**Positive and consistent use**. Positive connection made, either consciously or unconsciously between the equipment and the signal delivered. eg may request use, get upset if removed or not available, indicate when there is a fault.<br><br>*At Stage Five the child actively monitors their auditory environment for extended periods, may 'request' the equipment or demonstrate awareness of changes in signal quality, sufficient for this to affect behaviour.*
4	**Sustained behaviour changes** when the equipment is worn which demonstrate **functional use** of the signal for extended parts of the waking day.<br><br>*The move to Stage Four requires the child's overall attention skills and ability to take an interest in the world around to be sufficiently developed for them to independently monitor their auditory environment for at least part of the time. This is a huge challenge for PMLD children or those with an additional diagnosis of autism.*
3	**Definite behaviour changes** when equipment is worn although these may only be for highly motivating sounds/voices and within structured activities. Typically, daily/ regular use but may only be for part of the time.<br><br>*This is a broad category which encompasses children where sound processor use may be inconsistent, but benefit is evident during these periods, through to children who wear the equipment for most of their waking hours but whose spontaneous auditory monitoring abilities remain limited.*
2	**Passive acceptance** of the equipment and signal. Behaviour changes *not* evident. May have periods of extended use (ie as much as all waking hours).<br><br>*The equipment and signal is accepted but there are no evident behaviour changes. Not all children go through this stage and for others it may be quite a short period. However, for some children at this stage, wearing time may be for as much as most of their waking hours. This is because passive acceptance of the equipment and the signal, does not lead automatically to being able to recognise the information provided as meaningful. For many children within this population, maintaining attention is effortful and can only be achieved for limited periods for highly motivating objects, people and activities.*
1	**Limited use - short periods** - and continued episodes of non-use because signal is not tolerated. May only wear when distracted or occupied by adults.<br><br>*Equipment use is typically for short periods of time. At this stage the child is only likely to wear the equipment when there is a high level of adult attention, re-assurance and distraction. Without this, the child may try to knock off the coil etc.*
0	**No/little use** - equipment is rejected or actively removed. Adverse reaction to the signal<br><br>*As yet there is no effective use. The child is distressed or actively tries to remove the equipment. The physical sensation of the sound processor may be tolerated but only until the signal is activated.*
