# Supplementary material for: Translation, Cross-Cultural Adaptation and Preliminary Validation of the Italian Version of the Early Cognitive and Listening Links (EarlyCaLL) Framework
Source: Audiol Res. 2026 Oct 19;16(5):160. doi: 10.3390/audiolres16050160 (PMC13701395; doi:10.3390/audiolres16050160)
Supplement: Supplementary file 1 [file audiolres-16-00160-s001.zip › Supplementary S5.pdf]

Scala di valutazione delle abilità cognitive e comunicative precoce: EarlyCaLL

	SVILUPPO COGNITIVO	COINVOLGIMENTO SOCIALE ED EMOTIVO	ASCOLTO E COMPRENSIONE DELLE PAROLE	VOCALIZZAZIONE E PRIME PAROLE	AAC
10	**Utilizza un oggetto per rappresentarne un altro**. Gioco più organizzato. Guarda i dettagli sulle figure. Nota errori intenzionali nella normale routine. Inizia il gioco di immaginazione. Inizia a ordinare gli oggetti per categorie distinte, ad es colore, automobili vs treni	**Consapevolezza, comprensione e conformità alle prime regole sociali.** Qualche coinvolgimento attivo quando è in gruppo; gioca con gli altri o imita le loro azioni A volte partecipa in un piccolo gruppo.	**Segue una semplice conversazione per più di un turno di parola**. Risponde a semplici commenti e richieste degli adulti. Ascolta più a lungo e con un'attenzione più sostenuta. Ad esempio, se aiutato, può seguire un racconto molto semplice o raccontare un'esperienza condivisa.	**Inizia a conversare. Commenta in maniera autonoma.** Può porre semplici domande per ottenere informazioni	Combinazione di segni o simboli per esprimere e rispondere a semplici idee degli altri. Ad esempio sequenza di segni legati ad eventi quotidiani, bisogni, storie ripetute.
9	**Partecipa più attivamente alle routine quotidiane** – Riesce a ricordare una semplice sequenza di eventi. Nota i cambiamenti nella routine. Mostra più interesse per i libri. Può completare semplici attività al computer e risolvere semplici problemi.	**Partecipazione sostenuta all'interno di attività e routine familiari o divertenti con un'altra persona.** Ad esempio partecipare a canzoni e filastrocche. Può partecipare a semplici attività di cooperazione, ad esempio "dallo a..."	**Comprende le istruzioni con due parole chiave (senza supporto gestuale).** Segue le indicazioni, ad esempio dare la palla alla mamma. Sa rispondere in modo appropriato alle domande sulle esperienze molto recenti. fa uso di schemi ritmici in canzoni e di rime.	**Inizia la combinazione di parole**. Ha acquisito circa 50 parole. Usa singole parole con segni/ gesti / lallazione per ampliare il messaggio. Inizia a fare domande di base.	**Inizia a combinare segni o simboli**
8	**Riconosce gli oggetti chesi associano**. Può riconoscere fotografie/ disegni di oggetti comuni. Compie scelte semplici tra una serie di opzioni. Può utilizzare immagini (PECS) o altri mezzi, se necessario. Conosce le proprie cose e ricorda a chi appartengono gli oggetti.	**Partecipa a semplici sequenze di gioco interattive** Sequenze di gioco semplici. Giochi interattivi a turno con adulti, ad esempio indicando parti del corpo all'interno di un gioco.	**Risponde a una serie semplice di richieste e di istruzioni**. Conosce circa 50 parole. Capisce l'intonazione delle domande, ad esempio" Dov'è andata la mamma?"	**Usa almeno 10 parole/piccole frasi.** Imita frequentemente parte di ciò che ha ascoltato. Ripete espressioni familiari – *"finito tutto"*	**Usa una gamma crescente di segni o simboli**. Usa il gesto naturale a supporto dei segni.
7	**Si concentra intensamente per brevi periodi**. Collabora più attivamente in semplici routine. E' interessato a capire come le cose si associano tra loro. Può completare un puzzle di forma molto semplice o un programma simile al computer.	**Dimostra la comprensione precoce delle emozioni degli altri.** Ad esempio,si arrabbia quando il genitore piange. Su richiesta può accarezzare una bambola / un orsacchiotto "che piange". Il gioco può essere esteso da una persona familiare.	**Riconosce poche frasi di uso comune (una parola chiave) senza supporto gestuale o visivo. Conosce il nome nomi di alcuni oggetti.** Conosce un numero più ampio di parole e alcune frasi strettamente legate alla routine.	**Emergono prime parole insieme a suoni simbolici e lallazione.** Partecipa a canzoni familiari. Suoni simbolici nel gioco.	Comprende almeno 15 segni. Utilizza in modo espressivo circa 5 segni. Inizia a utilizzare alcuni oggetti di riferimento per fare richieste.
6	**Semplici compiti di risoluzione di problemi** Utilizza una sequenza semplice nella routine di gioco. Mostra quando vuole che un'azione si ripeta. Riconosce alcuni oggetti di uso quotidiano. Può deliberatamente far cadere o lanciare oggetti e guardarli a terra. Può consegnare un oggetto su richiesta.	**Attenzione congiunta stabilita**. Aumento dell'uso dei gesti per la comunicazione sociale, ovvero non solo attraverso l'utilizzo dell'adulto come uno strumento. Mostra deliberatamente quando vuole che un'azione si ripeta, ad esempio "di più" / "di nuovo". Saluta con la mano. Raddrizza e scuote la testa.	**Riconosce alcune parole molto significative in un contesto ristretto**. Può guardare o fissare lo sguardo quando viene pronunciato il nome di persone o di oggetti familiari. Riconosce onomatopee e le collega al giocattolo o all'animale appropriato. Comprende e risponde ad alcune parole chiave di ambito sociale ed emotivo. Muove la mano per fare "ciao" quando glielo si richiede. Capisce NO!	**Alcune vocalizzazioni riflettono l'accesso al suono.** Produce e imita semplici patterns di suono. Alcune vocalizzazioni spontanee sono prodotte con un suono più intonato.	**Comparsa dei primi segni con coerenza, riconoscibili alle persone familiari.** Può usare un'immagine o un simbolo per fare richieste. Cerca di imitare i segni, producendo movimenti simili o forme delle mani.
5	**Anticipa le routine familiari**. Può usare un oggetto familiare o un indizio per anticipare la richiesta. Comparsa della compresione della permanenza delle persone e degli oggetti. Sorride riconoscendosi allo specchio.	**Qualche iniziativa spontanea ed interazione sociale volontaria**, ad esempio, si allunga cercando di afferrare le cose, punta gli occhi su oggetti di interesse. Primi giochi di interazione. Segue lo sguardo di un altro o indica col dito. Ride se c'é qualcosa che lo sorprende. Può ripetere un'azione se ottiene una reazione da parte di altri.	**Mantiene l'ascolto abbastanza a lungo da collegare i suoni agli eventi (previsione semplice)** Ad esempio, il rumore di una porta che si apre, reagisce alle voci di persone fuori dal campo visivo. Reagisce in modo chiaro al proprio nome. L' ascolto è più sostenuto.	**Turno comunicativo stabilizzato**. La gamma di vocalizzazioni si allarga. Le prime consonanti compaiono nel gioco vocale. La voce ha una qualità più cantata. Ha stabilito un ciclo di feedback uditivo. Lallazione con combinazioni CV-CV, ad esempio ma-ma.	
4	**Indica una semplice preferenza, ad esempio rifiutando qualcosa di indesiderato**. Gli oggetti spostati al di fuori de campo visivo vengono rapidamente dimenticati. Esplora gli oggetti in diversi modi, prevalentemente attraverso la bocca e le mani. Può afferrare e tenere oggetti in entrambe le mani e può passare oggetti da una mano all'altra.	**Riconosce il nesso tra le cose e avvia la comunicazione ad un livello iniziale con persone familiari**. Ad esempio, mostra la preferenza di sguardo o di movimento nella direzione di una persona familiare. Può mantenere uno sguardo sostenuto / interesse per l'interazione sociale molto precoce. Può imitare le espressioni facciali.	**L'attenzione può essere attirata con la chiamata a voce e può essere sostenuta per brevi periodi**. Ascolta attivamente suoni e voci a distanza ravvicinata. Riconosce voci familiari. Risponde in modo diverso a voci sovrapposte o rassicuranti.	**Vocalizza con intento comunicativo**. Vocalizzazione intenzionale, ad esempio per ottenere attenzione, salutare persone conosciute, avviare la comunicazione.	**Mostra l'intento di comunicare semplici emozioni e/o bisogni**, attraverso movimenti del corpo, espressione facciale o vocalizzazioni. Guarda volti e mani con interesse
3	**Consapevolezza ed esplorazione precoci.** Afferra ed esplora gli oggetti - anche con la bocca, ecc. Inizia a comprendere dei nessi di causalità – ripete delle azioni deliberatamente. Allerta visiva: segue gli oggetti in movimento da una parte all' altra. Muove le mani dai lati alla linea mediana ed esplora le proprie mani e le proprie dita. Avverte e reagisce al cambiamento mentre si sposta in ambienti diversi.	**Risponde all'adulto attraverso il movimento, stando vicino, ecc**. Esprime emozioni molto semplici oltre il disagio. Gli adulti familiari riescono ad interpretare il significato di alcune vocalizzazioni / espressioni facciali / movimenti del corpo.	**Il suono ha un significato e/o una dimensione emotiva**. Alcune risposte positive ed negative a voci, musica o ai suoni ambientali più frequenti e che possono renderlo contento. Può divertirsi giocando con giocattoli che generano suoni e ripetendo l'azione per riprodurre il suono..	**Vocalizzo** Variazioni in volume e altezza. Può vocalizzare maggiormente con persone familiari. Può vocalizzare con loro.	**Coinvolgimento precoce**: guarda le attività e le persone
2			**Consapevolezza** Dimostra conoscenza di alcuni suoni a distanza ravvicinata / forte intensità. Può sorridere o essere più attento in risposta alla chiamata diretta. Risposte non ancora coerenti.	**Uso della voce.** Maggiore uso della voce.	
1	**Movimenti Riflessi** E' interessato ai volti e agli oggetti vicini al suo campo visivo	Movimenti riflessi. Reagisce al tatto.	**Detezione**. Semplici risposte riflesse ai suoni. Detezione fugace, non registrata come significativa.	**Semplici suoni riflessi.** Nessuna informazione attraverso l'udito	**Consapevolezza limitata degli altri**: comunicazione pre-intenzionale
0			**Nessuna risposta**		
