# Supplementary material for: Translation, Cross-Cultural Adaptation and Preliminary Validation of the Italian Version of the Early Cognitive and Listening Links (EarlyCaLL) Framework
Source: Audiol Res. 2026 Oct 19;16(5):160. doi: 10.3390/audiolres16050160 (PMC13701395; doi:10.3390/audiolres16050160)
Supplement: Supplementary file 1 [file audiolres-16-00160-s001.zip › Supplementary S6.pdf]

Nottingham Auditory Implant Programme

Nottingham University Hospitals NHS Trust

Appendix Two Early Cognitive and Listening Links: Early CaLL

	COGNITION	SOCIAL AND EMOTIONAL ENGAGEMENT	LISTENING, UNDERSTANDING WORDS	VOCALISATIONS, EARLY WORDS	AAC
10	**Uses one object to represent another.**<br>More organised play. Looks at the details on pictures.<br>Notices deliberate mistakes in the normal routine.<br>Small world play begins.<br>Begins to sort objects using a single distinctive feature.eg colour, cars v trains	**Awareness, understanding and some conformity to early social rules.**<br>**Some active group involvement; plays with others or copies their actions**<br>Sometimes participates in a small group.	**Follows a simple conversational exchange across more than one turn.**<br>Responds to simple adult comments and requests.<br>Listens for longer and with more sustained attention.<br>E.g. With support, can follow a very simple story or retell of shared experience.	**Begins conversations. Offers own comments spontaneously.**<br>Can ask simple questions to obtain information	Combinations of signs or symbols to express and respond to the simple ideas of others.<br>Eg sequence of signs linked to daily events, needs, retells.
9	**Participates more actively in daily routines - can remember a simple sequence of events.**<br>**Notices changes in the normal routine.**<br>More sustained interest in books. May complete computer-based early problem solving tasks.	**Sustained participation within familiar or enjoyable activities and routines with another person.** E.g. action songs and rhymes.<br>Can participate in simple co-operative activities e.g. *give it to…*	**Understands two key word Instructions [without gestural support].** Follows directions e.g. *give the ball to mummy.* Can respond appropriately to questions about immediate experiences. Enjoys rhythmic patterns in songs and rhymes.	**Beginning word combinations**<br>Has about 50 words. Uses single words with signs/ gestures/babble to extend the message.<br>Begins to ask very basic questions.	**Beginning to combine signs or symbols**
8	**Recognises objects which go together.**<br>Can recognise photographs/ drawings of common objects. Makes simple choices from a number of preferred options. Can use pictures (PECS) or other means if needed.<br>Knows own things and remembers where objects belong.	**Engages in simple interactive play sequences**<br>Simple play sequences<br>Interactive turn-taking games with adults. E.g. pointing to parts of the body within a game.	**Responds to a range of simple requests and instructions.**<br>Understands c. 50 words.<br>Understands question intonation *e.g. Where's mummy gone*?	**Uses at least 10 words/holophrases**<br>Frequently imitates fragments of what is heard. Copies familiar expressions –*all gone*,	**Uses a growing range of signs or symbols**<br>Uses natural gesture to support signs..
7	**Concentrates intently for short periods.**<br>Co-operates more actively in simple routines.<br>Interested in how things fit together. May complete a very simple shape board puzzle or similar computer programme.	**Demonstrates early understanding of the emotions of others**. Eg becoming upset when parent cries .On request may pat 'crying' doll/teddy. Play can be extended by a familiar person.	**Recognises a few familiar phrases, (one key word) without sign/visual prompts.**<br>**Understands some object names.**<br>Understands a wider number of words and some phrases closely tied to routines.	**Emerging first words together with symbolic sounds and babble**<br>May join in with parts of familiar songs.<br>Symbolic sounds in play.	Understands at least 15 signs<br>Uses c. 5 signs expressively.<br>Begins to use some objects of reference to request
6	**Simple Problem solving**<br>Enjoys a simple sequence in a play routine. Shows when they want an action repeating. Recognises some everyday objects by use.<br>May deliberately drop or cast objects and watch them fall. May hand over an object on request. .	**Joint attention established. Increased use of gestures to communicate socially** ie not just using adult as tool. Deliberately shows when wants an action repeating e.g. 'more' / 'again'.<br>Waves bye bye. Nods and shakes head.	**Recognises a few highly meaningful words in close context.**<br>May look or point when familiar people or objects are named. Recognises symbolic vocalisations and links them to an appropriate toy or animal.<br>Understands and responds to a few key social and emotional words. May wave on hearing request to say bye-bye. Understands NO!	**Some vocalisations reflect access to sound**<br>Produces and imitates simple patterns of sound<br>Some spontaneous vocalisations are more tuneful	**First consistent signs emerge, recognisable to familiar people**<br>Can use a picture or symbol to request<br>Attempts to copy signs, producing similar movement or handshapes.
5	**Anticipates familiar routines**.<br>**Can use a familiar object or context clue to anticipate.**<br>Understanding of people and object permanence emerging. Smiles at self in mirror.	**Some intentional social initiation and interaction** E.g. reaches out, eye points to things of interest. Early turn-taking games. Follows another's eye gaze or point. Laughs at something surprising. May repeat an action if they get a reaction from others.	**Sustains listening for long enough to link sounds to events. [simple prediction]**<br>E.g. sound of a door opening, searches for people heard out of eye line.<br>Reacts distinctively to own name.<br>More sustained listening.	**Vocal turn taking established**.<br>. Range of vocalisations widens. Early consonants emerge in vocal play. Voice has a more sing-song quality. Auditory feedback loop established. Babble with CV-CV combinations e.g. *ma-ma*	
4	**Indicates a simple preference** e.g. by rejecting something unwanted. Objects moved outside of visual range are quickly forgotten. Explores objects in different ways, predominantly by mouthing but also by handling. May grasp and hold objects in either hand and can pass objects from hand to hand**.**	**Shows recognition, attachment, and initiates communication at an early level, with familiar people.** Eg shows preference for looking or moving in the direction of familiar person.<br>May sustain eye contact / interest in very early social interaction. May imitate facial expressions.	**Attention can be gained with voice and can be held for short periods.**<br>Actively listens to sounds and voices at close range.<br>Recognises familiar voices.<br>Responds differently to cross or calming voices.	**Vocalises with Intent to communicate**<br>Vocalises purposefully e.g. to gain attention, to greet known people, to initiate communication.<br>.	**Shows intent to communicate simple emotions and/or needs**, through body movements, facial expression or vocal behaviours . May watch faces and hands with interest
3	**Early awareness and exploration**<br>Reaches and explores objects - mouthing etc.<br>Begins to make causal links - repeats actions deliberately.<br>Visually alert - tracks moving objects from side to side.<br>Brings hands together from side to midline and explores own hands and fingers.<br>Notices and reacts to change as moves into different environments.	**Responds to adult overtures by movement, maintaining proximity, etc.**<br>Expresses very simple emotions beyond discomfort.<br>Familiar adults can interpret the meaning of some vocalisations/ facial expressions/ body movements.	**Sound has meaning and/or an emotional dimension.**<br>Some positive likes and dislikes with regard to voices, music or environmental sounds which are more often repeatable. This may result in an increase in general contentment. Enjoys playing with noise making toys repeats action to get sound again.	**Vocal Exploration**<br>Variations in volume, pitch.<br>May be more vocal with familiar people.<br>May vocalise to themselves	**Early engagement-** watches activities and people
2			**Awareness**<br>Demonstrates awareness of some sounds at close range/raised levels. May smile or be more attentive in direct response to voice. Not yet consistent.	**Uses voice**<br>Increased use of voice.	
1	**Reflex movements**<br>Interested in faces and objects in close visual field.	**Reflex movements**<br>Responds to touch.	**Detection.** Simple reflex responses to sounds.<br>Fleeting detection, not registered as meaningful.	**Simple reflexive sounds**<br>Not informed by hearing.	**Limited awareness of others-** pre-intentional communication
0			**No Response**		

Copyright©NUH NHS Trust 2016. All rights reserved. Not to be reproduced in whole or part without the permission of the copyright owner
