# Supplementary material for: Translation, Cross-Cultural Adaptation and Preliminary Validation of the Italian Version of the Early Cognitive and Listening Links (EarlyCaLL) Framework
Source: Audiol Res. 2026 Oct 19;16(5):160. doi: 10.3390/audiolres16050160 (PMC13701395; doi:10.3390/audiolres16050160)
Supplement: Supplementary file 1 [file audiolres-16-00160-s001.zip › Supplementary S7.pdf]

Appendici da tre a sette

Appendice tre

Early Cognitive and Listening Links: EarlyCaLL
Esempi dettagliati per stringa

	CAPACITA' COGNITIVE
10	**Usa un oggetto per rappresentarne un altro.** Gioco più organizzato. Guarda i dettagli sulle figure. Nota gli errori intenzionali nelle normali attività quotidiane E' in grado di iniziare spontaneamente il gioco di immaginazione, ad esempio, il gioco delle bambole: fingere di mangiare, di dormire, ecc. E' in grado di giocare con giocattoli di macchine o piccole figure. Inizia a ordinare gli oggetti in base ad una singola caratteristica distintiva. Ad esempio, ordinare i giocattoli di mezzi di trasporto per trovare tutte le auto.
9	**Partecipa più attivamente alle attività quotidian - può ricordare una breve e semplice sequenza di eventi.** es. Richiede il suo giubbotto se vede un adulto che si prepara per uscire. Nota i cambiamenti della routine, ad es. Si arrabbia se le cose non avvengono come al solito. Ha un interesse più sostenuto per i libri. es. Può seguire una storia molto semplice di un libro illustrato ripetuta e ha preferenze per determinate storie, rime, ecc.
8	**Riconosce gli oggetti che si associano,** ad es. Può essere in grado di associare un cucchiaio ad una pietanza o un piatto fare giochi di abbinamento. Riconosce le proprie cose e ricorda dove è il posto degli oggetti, ad esempio i vestiti. Compie scelte semplici tra una serie di opzioni.
7	**Si concentra intensamente per brevi periodi escludendo tutto il resto.** Gioca in modo indipendente per un breve periodo ed essere assorto nell'attività. Mostra un interesse per piccoli oggetti e per i dettagli, ad esempio come gli oggetti si associano tra loro - completare un semplice puzzle o giochi simili al computer, montare e smontare i giocattoli. Sa come si gioca con determinati oggetti, ad esempio facendo rotolare una palla. Collabora più attivamente in semplici attività quotidiane, ad esempio vestirsi.
6	**Semplice risoluzione dei problemi.** Si diverte nell' eseguire semplici attività di routine di gioco, ad esempio costruire e distruggere torri di mattoncini, mettere oggetti dentro e fuori dai contenitori.<br>Dimostra di comprendere il meccanismo di causa-effetto per risolvere problemi, ad esempio tirare una corda per avvicinare un giocattolo. Fa vedere quando vuole che un'azione si ripeta, ad es. tiene la mano in posizione per ripetere il girotondo. Riesce a consegnare un oggetto su richiesta.<br>Riconosce alcuni oggetti della quotidianità in base all'uso e può riconoscere foto di oggetti, familiari, persone, animali.
5	**Anticipa le routine conosciute** È in grado di utilizzare un oggetto familiare o un indizio del contesto per anticipare una routine o un'attività conosciuta; ad esempio, mettersi in una posizione particolare per dimostrare di essere pronto per un'attività piacevole o un gioco. Inizia a piangere quando vede un pannolino se non gli piace essere cambiato. Anticipa un gioco vedendo un giocattolo che conosce.<br>**Comparsa della comprensione della permanenza dell'oggetto** es Reagisce con sorpresa quando un oggetto cade o scompare e guarda per vedere dove è andato. Ama i giochi a cucù. Aumenta la consapevolezza di sé, ad esempio, può sorridere quando si vede allo specchio.
4	Indica una semplice preferenza rifiutando qualcosa che non vuole. Può indicare l'oggetto preferito, ad esempio un alimento, raggiungendo o puntando lo sguardo, muovendosi di più. Esplora gli oggetti in diversi modi, prevalentemente con la bocca ma anche con le mani o con i piedi. Può afferrare e tenere oggetti con entrambe le mani e può passare oggetti da una mano all'altra; es. scuotere deliberatamente un sonaglio per emettere un suono. Gli oggetti spostati al di fuori del campo visivo vengono rapidamente dimenticati.
	**Consapevolezza ed esplorazione precoci** **Raggiunge ed esplora oggetti**, anche con la bocca, ecc. **Inizia a creare collegamenti causali** – ripete volontariamente azioni, ad es. scorre su un giocattolo mobile. **Segue con lo sguardo gli spostamenti** - sposta gli oggetti da un lato all'altro. Guarda brevemente da un oggetto all'altro. **Unisce una mano con l'altra** ed esplora le mani e le dita. Mantiene un oggetto per alcuni secondi quando gli é stato dato in mano.
2	**Avverte e reagisce al cambiamento** quando si sposta in ambienti diversi. Può essere più tranquillo in ambienti familiari.
1	**Comportamenti riflessi** Risponde a volti e oggetti in un campo visivo ravvicinato. Es. Apre la bocca per succhiare in risposta al tatto, gira la testa e gli occhi verso la fonte di luce o chiude gli occhi in condizioni di luce intensa, reagisce a diverse consistenze sulla pelle.

Copyright©NUH NHS Trust 2019. Tutti i diritti riservati. Da non riprodurre in tutto o in parte senza il permesso del titolare dei diritti d'autore

	COINVOLGIMENTO SOCIALE ED EMOTIVO
10	**Dimostra di comprendere e di avere atteggiamenti conformi a semplici regole sociali all'interno di piccoli gruppi.** Ad esempio, può partecipare attivamente per brevi periodi in un'attività di gruppo, osservando ciò che fanno gli altri e talvolta copiando le azioni degli altri. A volte può aspettare il suo turno o di essere interpellato, rispondere a ciò che gli altri vogliono che faccia o reagire contro questa cosa.
9	**Partecipazione sostenuta ad attività piacevoli sociali e familiari e di routine con un'altra persona.** Partecipa a canzoni e filastrocche mostrando consapevolezza dell'altra persona, partecipando all'attività fino alla fine della canzone, ecc. Può completare semplici attività di cooperazione come distribuire oggetti ad altri su richiesta o guardare gli altri mentre li ricevono e guardare tra l'oggetto e un'altra persona con consapevolezza del gesto.
8	**Si impegna in brevi e semplici sequenze di gioco interattive.** Giochi interattivi a turni con adulti, ad es. far rotolare una palla avanti e indietro, indicare parti del corpo su richiesta, fingere di mangiare, bere, dormire.
7	**Dimostra comprensione precoce delle emozioni degli altri.** Ad esempio, può spontaneamente preoccuparsi se qualcuno è visibilmente turbato e può provare a fare un semplice gesto di conforto. Ad esempio, su richiesta coccolare o accarezzare una bambola/orsacchiotto che "piange". Ottenere beneficio giocando a dare supporto/incoraggiamento di una persona familiare.
6	**Attenzione congiunta stabilita. Maggiore comprensione/uso dei gesti per la comunicazione sociale** Capisce che qualcun altro può aiutarlo a realizzare qualcosa che non può fare da solo. Si rivolge a qualcuno familiare per capire le loro risposte a un evento. Può offrire cose/condividere e dirigere l'attenzione di qualcuno su qualcosa, ad esempio indicare cose di interesse e successivamente controllare la reattività/reazione degli adulti, invece di usare un adulto per fare cose per se st~~esso. Capisce~~ e partecipa a suo modi ai saluti di tutti i giorni come saluatarecon la mano
5	**Qualche iniziativa sociale intenzionale per esprimere e condividere una serie di semplici bisogni, emozioni e interessi. Ad e**s. si avvicina, punta lo sguardo su cose di interesse, alza le braccia per chiedere di essere preso in braccio. Ride di qualcosa di sorprendente, esprime disagiose separato da persone familiari. Mostra quando vuole che un'azione si ripeta, ad es. prova a compiere le azioni per mostrare di volere di nuovo lo stesso gioco.
4	**Partecipa alla comunicazione ad un livello con persone familiari. Sorride con scopo sociale o una risposta di piacere simile e specifica al contatto con un'altra persona** Mostra maggiore reattività, affetto e interesse per alcune persone Familiari. Stabilisce un contatto visivo e si impegna per brevi periodi nelle prime interazioni sociali, ad esempio guardando/facendo rumori/sorridendo/muovendo braccia e gambe. Può trovare i volti particolarmente interessanti e guardare più attentamente le espressioni facciali esagerate e copiare inconsciamente.
3	**Risposte alle aperture degli adulti calmandosi, sorridendo, muovendosi, mantenendo la vicinanza, ecc.Esprime emozioni molto semplici** Può rispondere al tatto e alla presenza degli altri mediante cambiamenti comportamentali come calmarsi, rilassarsi. Può ridere con piacere durante le attività preferite. Può tornare dall'adulto familiare o appoggiarsi per ottenere conforto e rassicurazione emotiva e fisica. Gli adulti familiari possono interpretare il significato di alcuni comportamenti, ad esempio vocalizzazioni/espressioni facciali/movimenti del corpo per esigenze diverse.
2	
1	**Comportamenti riflessivi** Esprime la fame e il disagio attraverso il pianto.

Copyright©NUH NHS Trust 2019. Tutti i diritti riservati. Da non riprodurre in tutto o in parte senza il permesso del titolare dei diritti d'autore

	ASCOLTO E, COMPRENSIONE DELLE PAROLE
10	**Segue e partecipa ad un semplice conversazione per più di un turno.**<br>Risponde a una semplice serie di commenti e richieste di adulti. Ascolta più a lungo e con un' attenzione più sostenuta per brevi periodi. Ad esempio, se aiutato, può seguire una storia molto semplice o un libro illustrato o raccontare un'esperienza condivisa.
9	**Capisce le istruzioni con due parole chiave [senza supporto gestuale].**<br>Es. Segue le indicazioni costituite dalla comprensione di 2 elementi ad esempio *dare la palla alla mamma*.
8	**Risponde in modo appropriato a una serie di semplici richieste e istruzioni (1-2 parole chiave).**<br>Conosce circa 50 parole. Ad es. Battere le mani, indicare parti del corpo su richiesta come. "Dov'è il tuo naso?" Segue semplici istruzioni come "Dallo a papà", "Prendi le scarpe" o "Chiudi la porta".<br>Alla domanda "Dov'è il cane?" cerca visivamente poi guarda all'adulto puntando lo sguardo o usando la voce per rispondere.
7	**Riconosce una serie di frasi familiari (una parola chiave). Conosce il nome di alcuni oggetti.**<br>Conosce un numero più ampio di parole e alcune frasi strettamente legate alla routine,d es. Non toccare, andiamo,
6	**Riconosce un piccolo numero di parole altamente significative e suoni simbolici in un contesto chiuso.**<br>Può guardare o indicare quando vengono nominati persone o oggetti familiari, può collegare delle vocalizzazioni simboliche all'oggetto corretto, può provare a dire o salutare con la mano quando si chiede a voce di dire "ciao", può reagire negativamente alla parola "no".
5	**Rimane in ascolto abbastanza a lungo da riconoscere e collegare i suoni agli eventi e utilizzarlo per prevedere eventi molto familiari**<br>Cerca persone ascoltate fuori dal campo visivo e traccia i movimenti ascoltando, mostra attraverso un comportamento anticipatorio che ha riconosciuto alcuni suoni quotidiani significativi, come l'acqua che scorre nella vasca da bagno,i suoni della preparazione del cibo.
4	**L'attenzione può essere attirata e mantenuta con la voce per un breve periodo.**<br>Ascolta attivamente suoni e voci vicini a seconda del proprio livello di attenzione generale.<br>Riconosce voci familiari e in determinate situazioni può iniziare a riconoscere il proprio nome.<br>Dimostra preferenze per determinate canzoni e filastrocche, mostra risposte positive o avversione alle voci, alla musica o ai suoniambientali più frequenti.
3	**Il suono ha un significato e una dimensione emotiva.**<br>Può attribuire un significato a un numero limitato di suoni, in maniera sufficiente per assumere comportamenti che possono aumentare la soddisfazione generale. Es. voci familiari, musica rilassante. Può divertirsi giocando con oggetti/giocattoli che generano suoni e ripetendo l'azione per riprodurre il suono.
2	**Consapevolezza**<br>Dimostra conoscenza di alcuni suoni e della voce a distanza ravvicinata/a forte intensità. Può sorridere o diventare in breve più attento. Risposte non coerenti.
1	**Detezione**<br>Semplici risposte riflesse al suono.<br>Detezione fugace del suono ma non considerato significativo
0	**Nessuna risposta al suono**

Copyright©NUH NHS Trust 2019. Tutti i diritti riservati. Da non riprodurre in tutto o in parte senza il permesso del titolare dei diritti d'autore

Appendice sei

	VOCALIZZAZIONI E LINGUAGGIO PRECOCE
10	**Avvia la conversazione. Propone spontaneamente i propri commenti.**<br>Partecipa o avvia conversazioni semplici, ad esempio "Il mio bambino ha i capelli scuri". Può porre semplici domande per ottenere informazioni, ad esempio "Macchina in arrivo... a casa, mamma?"
9	**Prime combinazioni di parole**<br>Es. "Macchina della mamma". Dice più di 50 parole. A volte usa singole parole accompagnate da segni/gesti/balbettii percompletare il messaggio. Molte imitazioni degli altri. Può riferirsi a se stesso con il nome.
8	**Dice almeno 10 singole parole**<br>Imita frequentemente parte di ciò che ascolta. Ripete espressioni familiari : *"finito tutto".* Tenta di cantare. Partecipa alle filastrocche.
7	**Comparsa delle prime parole, insieme a suoni simbolici e alla lallazione**<br>Linguaggio parlato simile al vociare chatter. Per fare riferimento a qualcosa, usa lo stesso pattern sonoro in modo coerente, che potrebbe non corrispondere al pattern degli adulti.
6	**Produce e imita semplici pattern sonori**<br>Le vocalizzazioni spontanee sono più simili a quelle del parlato. Alcuni vocalizzi. Gli schemi di intonazione della lingua madre sono chiari. Può avere una lallazioneintensa e intonata per lunghe serie ripetitive di sillabe da-da, mam-ma-ma. Puòimitare i suoni dei giochi - schioccare le labbra, tossire. Può iniziare una lallazione variata di sillabe non ripetitive, ad es . *badadida.*
5	**Alcune vocalizzazioni riflettono l'accesso al suono**<br>Emergono alcune caratteristiche riconoscibili della lingua madre, ad esempio, la voce può sviluppare una qualità più cantilenante, la gamma di vocalizzazioni si amplia includendo vocali e consonanti dalla lingua madre. Può effettuare la lallazione con combinazioni CV-CV, ad esempio *ma-ma*
4	**Vocalizzazione a scopo comunicativo**<br>Può vocalizzare per attirare l'attenzione, per salutare persone conosciute, per iniziare la comunicazione. Può fare silenzio e prestare attenzione quando si parla e può vocalizzare di più nei giocih con una persona di fronte.. Può rifiutare e richiedere usando la voceinsieme alle espressioni facciali o ai movimenti del corpo. La vocalizzazione tende ad essere una serie di suoni simili, prevalentemente vocalici, che non assomigliano ancora alle vocali e alle consonanti della lingua madre.
3	**Esplorazione vocale**<br>Presenza di variazioni in volume ed in altezza. Produce priincipalmente di suoin di tipo vocalico.. Ride, ridacchia e grida infastidito. Produce principalmente suoni vocalici aperti o suoni che danno un feedback tattile, ad esempio rumori gutturali, soffiare bolle
2	
1	**Suoni riflessivi**<br>ad es. pianto, eruttazione

Copyright©NUH NHS Trust 2019. Tutti i diritti riservati. Da non riprodurre in tutto o in parte senza il permesso del titolare dei diritti d'autore

Appendice sette

	COMUNICAZIONE AUMENTATIVA E ALTERNATIVA (AAC)
10	**Trasmette idee e informazioni semplici utilizzando combinazioni di segni, simboli o parole scritte** Può partecipare alla conversazione.
9	**Inizia a combinare insieme segni, simboli o parole scritte.**<br>Ad esempio, produce spontaneamente il gesto "bimbo che piange" o risponde a domande come "Dov'è papà?"<br>Nelle attività strutturate, combina i simboli per "volere" e "computer", puntando lo sguardo sull'immagine scelta o posizionando i simboli in sequenza su una lavagna.
8	**Utilizza una gamma crescente di singoli segni, simboli o parole scritte (almeno 10).**<br>I segni riflettono le forme di base della mano con movimenti semplici (ad es. avanti, su o giù) - sono riconoscibili ma possono essere ancora immaturi.<br>Usa un gesto naturale e il mimo per supportare i segni.
7	**Utilizza segni singoli, immagini, simboli o parole scritte**<br>Compaiono segni coerenti, riconoscibili da persone familiari. Per porre delle richieste può usare un'immagine o un simbolo (possibilmente all'internodel PECS), l'attenzione diretta e il commento.
6	**Acquisizione dei prerequisiti alla comunicazione.**<br>Utilizza una gamma più ampia di gesti e indica di più con lo sguardo. Compaiono alcuni iniziali segni/gesti sociali iconici, ad esempio il saluto con la mano, scuotere la testa per dire "no". Potrebbe comparire il manual babbling.<br>Inizia ad utilizzare oggetti di riferimento ad esempio può dare la tazza ad un adulto per richiedere da bere.<br>Indica o indirizza lo sguardo per indicare oggetti di interesse o richiedere un oggetto, ad esempio indica la foto di un genitore e sorride, oppure indirizza lo sguardo per indicare un'attività che gli piace.
5	
4	**Mostra l'intenzione di comunicare attraverso i movimenti del corpo, l'espressione facciale e gesti semplici,** ad esempio rifiuta spingendo via il cibo non desiderato, alza le braccia o punta gli occhi verso un giocattolo che vuole. Puòguardare selettivamente le mani, in particolare negli ambienti in cui i segni sono usati frequentemente.
3	**Impegno anticipato per brevi periodi**<br>Mostra interesse per le persone, in particolare per i volti.
2	
1	**Comportamenti non intenzionali** che gli adulti devono interpretare per soddisfare i bisogni precoci del bambino, ad esempio piange o piagnucola per richiedere cibo o il cambio del pannolino. Potrebbe avere reazioni al tatto.

Copyright©NUH NHS Trust 2019. Tutti i diritti riservati. Da non riprodurre in tutto o in parte senza il permesso del titolare dei diritti d'autore
