# Supplementary material for: Translation, Cross-Cultural Adaptation and Preliminary Validation of the Italian Version of the Early Cognitive and Listening Links (EarlyCaLL) Framework
Source: Audiol Res. 2026 Oct 19;16(5):160. doi: 10.3390/audiolres16050160 (PMC13701395; doi:10.3390/audiolres16050160)
Supplement: Supplementary file 1 [file audiolres-16-00160-s001.zip › Supplementary S8.pdf]

Early Cognitive and Listening Links: Early CaLL

Appendices three to seven

Detailed examples for strands

Appendix three

	COGNITION
10	**Uses one object to represent another.** More organised play. Looks at the details on pictures. Notices deliberate mistakes in the normal routine. Small world play may begin eg doll play –feeding, sleeping games etc. May play with transport toys or small figures. May begin to sort objects using a single distinctive feature. Eg Can sort transport toys to find all the cars,
9	**Participates more actively in daily routines - can remember a short, simple sequence of events.** eg May want own coat if sees adult getting ready to go out. Notices changes in the usual routine eg Gets upset if things don't happen as usual. More sustained interest in books. eg Can follow a very simple repeated picture book story and has preferences for certain stories, rhymes etc.
8	**Recognises objects which go together** eg May be able to link spoon with a dish/plate or play matching games. Knows own things and remembers where objects belong eg clothing. Makes simple choices from a number of options.
7	**Concentrates intently for short periods to the exclusion of all else.** May play independently for a short time and get absorbed in the activity. May show an Interest in small objects and details, eg how things fit together- complete a very simple shape board puzzle or similar computer programme, linking/pulling apart toys. Knows how to play specifically with certain objects.eg rolling a ball. Co-operates more actively in simple routines eg dressing.
6	**Simple problem solving.** Enjoys a simple play routine eg building and knocking down towers of brick, taking things in and out of containers. Demonstrates understanding of cause-effect to solve problems eg pulling a string to bring a toy closer. Shows when they want an action repeating eg keeps hand in place for repeat of round and round the garden. May hand over an object on request Recognises some everyday objects by use and may recognise photos of familiar objects, people, animals.
5	**Anticipates familiar routines.** Can use a familiar object or context clue to anticipate a familiar routine or activity. eg May get into a particular position to show they are ready for an enjoyed activity or game. Starts to cry when sees a nappy if they don't like being changed Anticipates a game from seeing a familiar toy. **Understanding of object permanence emerging** eg Reacts with surprise when an object falls or disappears and looks to see where it has gone. Enjoys peek-a-boo games. Growing self-awareness eg may smile at self in mirror.
4	Indicates a simple preference by rejecting something unwanted. May indicate the preferred object eg food item by reaching or eye pointing, becoming more animated. Explores objects in different ways, predominantly by mouthing but also using hands or feet. May grasp and hold objects in either hand and can pass objects from hand to hand. eg May shake a rattle deliberately to make a sound Objects moved outside of visual range are quickly forgotten.
3	**Early Awareness and Exploration** **Reaches and explores objects** - mouthing etc. **Begins to make causal links** - repeats actions deliberately eg swipes at a mobile toy **Tracks visually** - moving objects from side to side. Looks briefly from one object to another. **Brings hands together from side to midline** and explores own hands and fingers. May hold an object for a few seconds if placed in hand. **Notices and reacts to change** as moves into different environments. May be more settled in familiar environments.
2	
1	**Reflex behaviours** Responds to faces and objects in close visual field.eg Opens mouth to suck in response to touch, turns head and eyes towards light source or closes eyes in bright lights, reacts to different textures against skin.

Copyright©NUH NHS Trust 2019. All rights reserved. Not to be reproduced in whole or part without the permission of the copyright owner

Appendix four

	**SOCIAL AND EMOTIONAL ENGAGEMENT**
**10**	**Demonstrates some understanding and conformity to simple social rules within small group participation.** Eg Can actively participate for short periods within a group activity, taking some note of what others do and sometimes copying the actions of others. Can sometimes wait for a turn, wait for their name, respond to what others want them to do or react against this
**9**	**Sustained participation within familiar and enjoyable social activities and routines with another person.** Takes part in action songs and rhymes showing an awareness of the other person, staying with the activity until the end of the song etc. Can complete simple co-operative activities such as giving out objects to others on request or watching them being given and looking between the object and another person with understanding.
**8**	**Engages in short, simple interactive play sequences** Interactive turn-taking games with adults eg rolling a ball back and forth, pointing to parts of the body on request, pretending to eat, drink, sleep.
**7**	**Demonstrates some early understanding of the emotions of others.** Eg May spontaneously look concerned if some-one is visibly upset and may try to make a simple gesture of comfort e.g. On request may cuddle or pat a 'crying' doll/teddy. Obtains benefits in play from the support/encouragement of a familiar person.
**6**	**Joint attention established. Increased understanding/use of gestures to communicate socially** Understands that some-one else can help them to achieve something they can't do alone. Looks to some-one familiar to gauge their responses to an event. May offer things / share and directs someone's attention to something eg point to things of interest and then check back socially on adult responsiveness/reaction. Ie more than using adult to do things for them. Understands and participates in own way for everyday social greetings such as waving bye bye
**5**	**Some intentional social initiation to greet and convey a range of simple needs, emotions and interests** E.g. reaches out, eye points to things of interest, lifts arms to request to be picked up. Laughs at something surprising, expresses distress if separated from familiar people. Shows when wants an action repeating e.g. may attempt the actions to show they want the same game again
**4**	**Participates in communication at a simple level with familiar people. Has a social smile or similar, specific pleasure response to contact with another person** Shows increased responsiveness, affection and interest in a few key people. Makes eye-contact and engages for short periods in early social interactions eg by looking/ making noises/smiling/moving arms and legs. May find faces particularly interesting and look more intently at exaggerated facial expressions and unconsciously copy.
**3**	**Responses to adult overtures by calming, smiling, movement, maintaining proximity, etc. Expresses very simple emotions** May respond to touch and the presence of others by behaviour changes such as calming, quietening, relaxing. May laugh with pleasure during favourite activities. May return to familiar adult or lean in to get emotional and physical comfort and reassurance. Familiar adults can interpret the meaning of some behaviours eg vocalisations/ facial expressions/ body movements for different needs.
**2**	
**1**	**Reflexive Behaviours** Expresses hunger and discomfort through crying..

Copyright©NUH NHS Trust 2019. All rights reserved. Not to be reproduced in whole or part without the permission of the copyright owner

Appendix five

	**LISTENING, UNDERSTANDING WORDS**
**10**	**Follows and participates in a simple conversational exchange across more than one turn.** Responds to a simple series of adult comments and requests. Listens for longer and with more sustained attention for short periods. E.g. With support, can follow a very simple story or picture book or retell of shared experience.
**9**	**Understands two key word Instructions [without gestural support].** Eg Follows directions in involving understanding 2 elements e.g. *give the ball to mummy.*
**8**	**Responds appropriately to a range of simple requests and instructions (1-2 key words).** Understands c. 50 words. eg Claps hands, points to parts of body on request e.g. Where's your nose? Follows simple instructions such as 'Give it to daddy' 'Get your shoes' or 'Shut the door'. When asked 'Where's the dog?' searches visually then looks to adult with point or vocalisation to respond.
**7**	**Recognises a range of familiar phrases, (one key word) Understands some object names.** Understands a wider number of words and some phrases closely tied to routines eg Don't touch, let's go,
**6**	**Recognises a small number of highly meaningful words and symbolic sounds in close context.** Eg May look or point when familiar people or objects are named, may link symbolic vocalisations to the correct object, may try or wave on hearing request to say bye-bye, may react negatively to the word no
**5**	**Sustains listening for long enough to recognise and link sounds to events and use this to predict very familiar events** E.g. searches for people heard out of eye line and tracks movements by listening, shows through anticipatory behaviour that they recognise some meaningful everyday sounds such as the bath water running, sounds of food preparation.
**4**	**Attention can be gained and can be held with voice for a short period.** Actively listens to sounds and voices at close range in line with their general attention level. Recognises familiar voices and in certain situations, may begin to recognise own name. Demonstrates preferences for certain songs and rhymes, has positive likes and dislikes with regard to voices, music or environmental sounds which are more often repeatable.
**3**	**Sound has meaning and an emotional dimension.** Can attach meaning to a small number of sounds, sufficient to make a difference to aspects of behaviour which may result in an increase in general contentment. Eg familiar voices , calming music. May enjoys playing with noise making objects/toys will repeat action to make sound again.
**2**	**Awareness** Demonstrates awareness of some sounds and voice at close range/raised levels. May smile or briefly become more attentive. Responses not consistent.
**1**	**Detection** Simple reflex responses to sound Fleeting detection of sound but not registered as meaningful .
**0**	**No Response to sound**

Copyright©NUH NHS Trust 2019. All rights reserved. Not to be reproduced in whole or part without the permission of the copyright owner

Appendix six

	VOCALISATIONS & EARLY TALK
**10**	**Initiates conversational exchanges. Offers own comments spontaneously.**<br>Joins in or starts simple conversations.eg "My baby have brown hair." Can ask simple questions to obtain information eg "Taxi coming soon..... home-time mummy?"
**9**	**Beginning to combine words**<br>Eg "Mummy car". Has more than 50 words. Sometimes uses single words together with signs/ gestures/babble to extend the message. Lots of imitation of others. May refer to self by name.
**8**	**Has at least 10 single words**<br>Frequently imitates fragments of what is heard. Copies familiar expressions –*all gone*, Attempts to sing. Joins in nursery rhymes.
**7**	**Emerging first words, together with symbolic sounds and babble**<br>Speech-like chatter. Uses the same sound pattern consistently to refer to something. This may not resemble the adult model.
**6**	**Produces and imitates simple patterns of sound**<br>Spontaneous vocalisations are more speech-like. Some word-like vocalisations. The intonation patterns of the home language are clear. May babble loudly and tunefully in long repetitive strings of syllables da-da, mam-ma-ma. May imitates play sounds - lip smacking, cough May start to use non-repetitive babble strings eg *badadida.*
**5**	**Some vocalisations reflect access to sound**<br>Some recognisable features of home language emerge. eg voice may develop a more sing-song quality, range of vocalisations widens including vowels and consonants from the home language. May babble with CV-CV combinations e.g. *ma-ma*
**4**	**Vocalisation with intent to communicate**<br>May vocalise to gain attention, to greet known people, to initiate communication. May quieten and pay attention when spoken and may be more vocal in face to face play. May refuse and request using voice alongside facial expressions or body movements. Vocalisation tends to be a series of the same sounds, predominantly vowels. These do not yet sound like the vowels and consonants of the home language.
**3**	**Vocal exploration**<br>Demonstrated by variations in volume and pitch. Mainly vowel-like vocalisations. Laughs, chuckles and screams with annoyance. Mainly open vowel-like sounds or sounds that give tactile feedback eg guttural noises, blowing bubbles
**2**	
**1**	**Reflexive sounds**<br>eg crying, burping,

Copyright©NUH NHS Trust 2019. All rights reserved. Not to be reproduced in whole or part without the permission of the copyright owner

Appendix seven

	ALTERNATE AND AUGMENTATIVE COMMUNICATION (ACC)
**10**	**Conveys simple ideas and information using combinations of signs, symbols or written words**<br>Can participate in conversational exchanges.
**9**	**Begins to combine signs, symbols or written words together.**<br>Eg Spontaneously signs 'Baby cry' or responds to questions such as 'Where's Daddy?'<br>In structured activities, combines symbols for 'want' and 'computer', through eye pointing from a choice or by placing symbols in a sequence on a board.
**8**	**Uses a growing range of single signs,symbols or written words (at least 10).**<br>Signs reflect basic hand shapes with simple movements (eg straight forward, up or down) - are recognisable but may be still immature.<br>Uses natural gesture and mime to support signs.
**7**	**Uses single signs, pictures, symbols or written words**<br>Consistent signs emerge, recognisable to familiar people Can use a picture or symbol to request (possibly within PECS), direct attention, and comment.
**6**	**Pre-formal communication established.**<br>Using a wider range of gesture and more pointing. Some iconic or early social signs/gestures emerging eg waves bye-bye, shakes head for 'no'. Hand babble may emerge.<br>Beginning to use objects of reference eg may give cup to adult to request a drink.<br>Points or eye points to indicate objects of interest or request an object eg points to photo of parent and smiles or eye points to drawing of activity which he enjoys.
**5**	
**4**	**Shows intent to communicate through body movements, facial expression and simple gestures** eg refuses by such as pushing away food they do not want, raises arms or eye points towards a toy they want. May selectively watch hands, particularly in environments where sign is frequently used.
**3**	**Early engagement for brief periods**<br>Shows interest in people, particularly faces.
**2**	
**1**	**Unintentional behaviours** which adults to interpret to meet child's early needs eg if crying or 'grizzly' may need food or nappy change. May be responsive to touch.

Copyright©NUH NHS Trust 2019. All rights reserved. Not to be reproduced in whole or part without the permission of the copyright owner
