# Supplementary material for: Translation, Cross-Cultural Adaptation and Preliminary Validation of the Italian Version of the Early Cognitive and Listening Links (EarlyCaLL) Framework
Source: Audiol Res. 2026 Oct 19;16(5):160. doi: 10.3390/audiolres16050160 (PMC13701395; doi:10.3390/audiolres16050160)
Supplement: Supplementary file 1 [file audiolres-16-00160-s001.zip › Supplementary S9.pdf]

Dettagli paziente

Cognome	Nome	Data di nascita

Scheda del paziente

		Punteggi								
Data del test	Commenti della famiglia/care giver	Uso del dispositivo/ Tolleranza del segnale	Capacità cognitive	Coinvolgimento socio-emotivo	Ascolto, comprensione	Vocalizzazione, prime parole	AAC	Commenti	Nome e cognome del valutatore	Firma
